# The Influence of Perceived Overqualification on Cyberloafing: A Moderated Mediation Model of Frustration and Opportunities for Development

**DOI:** 10.3390/bs15121598

**Published:** 2025-11-21

**Authors:** Po-Chien Chang, Qingzi Jiang

**Affiliations:** School of Business, Macau University of Science and Technology, Macau SAR 999078, China; pcchang@must.edu.mo

**Keywords:** perceived overqualification, cyberloafing, frustration, opportunities for development, COR

## Abstract

Perceived overqualification represents a persistent structural challenge in contemporary labor markets and has garnered increasing scholarly attention in organizational behavior research. Informed by COR theory, we examine the psychological mechanism through which perceived overqualification leads to cyberloafing, introducing frustration as a mediator in this relationship. Furthermore, the moderating effect of opportunities for development on this indirect relationship is investigated. A two-stage questionnaire survey was conducted at a two-week interval, using a sample of 301 employees from ten companies in Guangdong and Guangxi, China. The results indicate that employees who view themselves as overqualified adopt cyberloafing by heightening the feeling of frustration. Notably, opportunities for development attenuate the indirect effect of perceived overqualification on cyberloafing via frustration. These findings contribute to the theoretical understanding of resource dynamics among overqualified employees and offer practical implications for the effective deployment and engagement of surplus talent in organizations.

## 1. Introduction

Overqualification, defined as a situation where employees’ knowledge, skills, abilities, experience, and/or education exceed job requirements (Erdogan et al., 2011; Maynard et al., 2006), has emerged as a pressing labor market issue in economies with rapid educational expansion. In China, the escalating number of university graduates starkly contrasts with limited high-skill job creation, intensifying this mismatch (Mo et al., 2023). Recent data indicate that the incidence of overeducation has consistently exceeded 30%, coupled with an education–job matching rate below 50%, leaving more than half of the Chinese workforce underemployed (Shanghai University of Finance and Economics, 2023). As growing graduates are now forced to seek positions that conventionally require lower qualifications, understanding the consequences of overqualification and mitigating its adverse effects has become imperative.

Increasing empirical evidence shows that individuals who possess excessive qualifications tend to exhibit various forms of workplace dysfunction, such as withdrawal in the workplace (Erdogan & Bauer, 2009; F. Liu et al., 2023), withholding of knowledge (J. Khan et al., 2022a; Ren et al., 2025), counterproductive behavior (S. Liu et al., 2014; Luksyte et al., 2011; Wiegand, 2023), and interpersonal abuse (S. Andel et al., 2022). One particular behavior of interest is cyberloafing, which refers to employees utilize organizational network resources during work hours for personal purposes (Lim, 2002). Although Some literature suggests that cyberloafing is negatively associated with firm performance and organizational productivity (Lim & Chen, 2012; Tandon et al., 2022). Earlier studies have found that there are certain benefits for employees who adopt cyberloafing, including enhanced mental status (J. Wu et al., 2020), stress relief (Kwala & Agoyi, 2025), and increased job satisfaction (X. Zhang et al., 2024).

However, there is evidence of the limitations in current studies examining how perceived overqualification influences cyberloafing in their theoretical approach. Equity theory underlines the fact that overqualified employees compensate for their perceived lack of justice by acting in a negative manner towards their organization (Cheng et al., 2020). The person-job fit literature argues that the employee’s job skills being less compatible with the job requirements leads to their needs being unmet, thereby hindering their work engagement (S. Andel et al., 2022). These approaches emphasize how overqualified employees recognize the external environment and regard cyberloafing as a means to respond to an unfavorable working environment, which significantly overlooks the possibility that overqualified individuals may resort to cyberloafing for the motivation of resource protection. In other words, current studies have paid little attention to how overqualified employees perceive their personal resources and how this perception affects their attitudes and behaviors (Shang et al., 2024).

The implications of the Conservation of Resources (COR) theory relate to the fact that humans are naturally motivated to protect and safeguard valued resources, as both actual and potential resource loss can cause psychological stress (Hobfoll, 1989). Notably, the paradox of overqualification is that employees hold ample capabilities but lack opportunities to apply them (Jiang et al., 2024; Shang et al., 2024; X. Wu & Ma, 2023). This situation reflects a waste of resources, which may strengthen individual motivation and behavior to protect their resources (Hobfoll et al., 2018). From this theoretical perspective, for overqualified employees, cyberloafing is more like a resource recovery mechanism, instead of a struggle against the external environment. Previous studies have preliminarily supported this assumption. Peng et al. (2023) indicate that employees adopt cyberloafing to cope with role stress, thus reducing resource consumption. Moreover, X. Zhang et al. (2024) regard cyberloafing as an effective way to attenuate workload and prevent further resource exhaustion. Thus, COR theory offers a compelling theoretical approach to uncover the underlying motivations driving overqualified employees to undertake cyberloafing.

COR theory further states that the loss of one type of resource can be compensated for by gaining resources of equal value in another domain (Hobfoll, 2001). This principle is extended to say that job opportunities for personal development, as one of the core resources, help reduce the positive effect of perceived overqualification on cyberloafing. Bakker and Bal (2010) define opportunities for development as assigned tasks that include learning opportunities, developing competencies, and growing personally. Accordingly, COR theory suggests that development prospects may fulfill the innate needs for personal growth (Rego & Cunha, 2009) that directly utilize the excess resources of the overqualified employees in question, decreasing the perception of potential resource loss. Previous studies have verified that developmental opportunities are important to workers’ attitudes toward overqualification (Erdogan et al., 2011). They also serve as the main situational factor reducing the adverse effects of overqualification (Rafiei & Van Dijk, 2024). Therefore, opportunities for development could be used as a mechanism for overqualified employees to minimize resource waste and decrease their frustration, which in turn leads to diminishing cyberloafing.

Thus, this article applies COR theory to shed light on the mechanism linking perceived overqualification to cyberloafing and the contextual factors underlying this relationship, distinguishing frustration and opportunities for development as the mediating emotion and the buffering factor, respectively. This study extends the research in organizations’ behavior through three main stages. Firstly, it proposes re-framing cyberloafing as a resource recovery mechanism for overqualified individuals under COR theory. This stance considerably differs from conventional interpretations based on equity theory (Cheng et al., 2020; J. Khan et al., 2022a) or the person-job fit model (S. Andel et al., 2022). This is because it is based on the dynamics of resources, which have been previously ignored in the mentioned frameworks. Secondly, this study finds that frustration is a vital emotional mediator, filling a gap between previous studies that emphasized either anger (J. Khan et al., 2022a) or boredom (S. Andel et al., 2022). The integration of COR theory and the frustration literature provides an empirical foundation for the assertion that restricted resource use engenders cyberloafing as a resource-replenishing behavior because it helps reduce psychological strain. Thirdly, this research underlines the role of job context in the overqualification conversation by supporting the development of a moderating buffer. Boundary conditions were examined to investigate the effect of perceived overqualification on employee behaviors (Shang et al., 2024; X. Wu & Ma, 2023); our findings validate the hypothesis that access to developmental opportunities is a moderating factor, appeasing frustration and curbing cyberloafing. This is a new contribution to COR theory, emphasizing the process of resource restoration.

## 2. Literature Review and Hypothesis Development

### 2.1. Perceived Overqualification and Cyberloafing

Cyberloafing is a behavioral phenomenon wherein employees voluntarily engage in non-work-related activities during work hours, using the organization’s digital means for purely personal purposes like browsing the internet, purchasing goods online, or occupying oneself with social media (Lim, 2002; Lim & Chen, 2012). Moreover, this practice consumes work attention (Lim, 2002) that might otherwise have been used for work-related tasks (Peng et al., 2023), leading to prolonged task completion (Lavoie & Pychyl, 2001), which might result in reduced fatigue and exhaustion (Wong et al., 2023; J. Wu et al., 2020). While the body of evidence in this field has relied on individual-level variables—like an individual’s traits (Jia et al., 2013), demographic characteristics (Garrett & Danziger, 2008), and habitual behavior patterns (Betts et al., 2014)—as well as organizational-level variables such as job design (Elrehail et al., 2021), leadership characteristics (Bhattacharjee & Sarkar, 2024; Peng et al., 2023), and organizational structure (Liberman et al., 2011), few studies have focused on how individuals perceive resource adequacy within themselves and how it affects their behavior.

COR theory indicates that individuals who experience or anticipate actual or perceived resource loss feel negative stress emotions and engage in resource protection behavior (Hobfoll, 1989; Hobfoll et al., 2018). Overqualification is one of the major indicators of personal resource waste (Jiang et al., 2024), since valuable resources obtained through a significant investment (such as education, work experience, skills, etc.) cannot be utilized in the present job, and then individuals perceive a sense of potential resource loss (Erdogan et al., 2011). In this context, cyberloafing permits overqualified members to avoid labor for an instant (J. Wu et al., 2020), during which they seize an opportunity to protect their resources (Peng et al., 2023; Tsai, 2023). As a result, cyberloafing may be a frequently adopted behavior by overqualified employees whose resources are undervalued (Cheng et al., 2020). In addition, workload underutilization is another characteristic of overqualification, where employees are given trivial tasks that require little or no effort (S. Liu et al., 2014; Sánchez-Cardona et al., 2020; Zhao & Ma, 2023). These limitations on resource utilization prohibit not only the use of in-hand resources but also the acquisition of new ones, which in turn forces compensatory investments against the perception of potential resource depletion (Zhao & Ma, 2023). This dynamic drives cyberloafing to minimize work-related resource expenditure and conserve personal resources (Tsai, 2023). Compared to other workplace deviances, cyberloafing can be performed directly at one’s workstation with lower detection risk and higher social acceptance (Bhattacharjee & Sarkar, 2024; Lim, 2002; X. Zhang et al., 2024). As a result, it can be considered an adaptive strategy for resource regulation among overqualified employees. Hence, we propose the following hypothesis:

**H1.**  *Perceived overqualification is positively related to cyberloafing.*

### 2.2. The Mediating Role of Frustration

Frustration is a negative psychological state that arises when individuals’ effective actions and motivations are obstructed or interfered with by various factors during purposeful activities (Fox & Spector, 1999; Spector, 1978). In organizational contexts, frustration often stems from work-related constraints such as painful job characteristics, limited promotion opportunities, role ambiguity, organizational changes, physical isolation, and job insecurity (Spector, 1978). These restrictive work conditions increase job difficulty by lowering the ceiling of job performance and disrupting employees’ emotional well-being (Strunk et al., 2022). Previous literature demonstrates that frustration experienced by employees is associated with undesirable resource conditions in the workplace. Within this line of work, the inappropriate utilization and reduction of resources are described as situations that can be linked to frustration. A. N. Khan et al. (2024) claim that abusive supervision largely drains employees’ job resources through improper work assignments, insufficient support, and unfair requirements, which contribute to job frustration. Similarly, Asim et al. (2025) argue that under exploitative supervision, employees’ physical and mental resources are constantly depleted, making them more prone to develop frustration. Inaddition, frustration occurs when individuals lack the necessary resources to achieve their goals or when their needs remain unmet (Eissa & Lester, 2017). Zia et al. (2025) further propose that the inability to accumulate resources to complete the work also constitutes a source of frustration.

According to COR theory, individuals are inherently motivated to retain and accumulate valued resources, and they are sensitive to current or future loss of resources (Hobfoll et al., 2018). We argue that being overqualified constitutes a form of resource wastage that generates employees’ perceptions of potential resource loss, which may increase frustration. Specifically, overqualification manifests a situation where employees’ skills, knowledge, and potential fail to be fully utilized in their current position, leading to inefficient resource utilization. Additionally, overqualification limits employees’ opportunities to obtain additional resources, thereby constraining their capacity to invest in and accumulate these over time (Zhao & Ma, 2023). These restrictive conditions tend to make frustration a more probable emotional response to overqualification (Eissa & Lester, 2017). Furthermore, the substantial gap between expectations and reality caused by overqualification undermines employees’ confidence in their own abilities (Wiegand, 2023) and diminishes their self-worth and psychological fulfillment (Sánchez-Cardona et al., 2020), which may drive a sense of frustration.

Moreover, according to Hobfoll et al. (2018), the feeling of frustration makes overqualified employees remain in a resource-depleted state, which in turn compels them to adopt protection strategies to save their remaining resources and prevent any further loss. We further suggest that overqualified employees who report high frustration are prone to engage in cyberloafing. Research found that deviant behavior can help mitigate the negative consequences of frustration (Azeem et al., 2021), as individuals tend to shift toward activities that are easy to conduct and can restore resources to reduce frustration (González-Gómez & Hudson, 2024). Cyberloafing, as a low-cost deviant behavior, enables employees to distance themselves from annoying situations, at the same time, distract themselves so as to recoup their exhausted resources (J. Wu et al., 2020). In particular, social media interactions provide psychological and interpersonal support, and build up emotional and cognitive resources. By visiting online news sites and watching bite-sized videos, employees obtain a brief respite, which facilitates resource recovery (Syrek et al., 2017). In addition, the concealment of cyberloafing is relevant because it gives room for employees to undertake such behavior without significantly affecting their performance, thus ensuring that the organization does not detect or punish them (Bhattacharjee & Sarkar, 2024). In sum, we contend that perceived overqualification is associated with cyberloafing through frustration as an emotional pathway. Based on this rationale, we formulate the following hypothesis:

**H2.**  *Frustration mediates the relationship between perceived overqualification and cyberloafing.*

### 2.3. The Moderating Role of Development Opportunities

Job resources stem from physical, psychological, social, and organizational domains, contributing to employee motivation and well-being and enabling the completion of workplace tasks (Bakker et al., 2004). These resources help buffer job demands, mitigate associated mental and physical strain, and facilitate goal achievement, personal growth, and continuous learning (Bakker & Bal, 2010). Among the most critical job resources are autonomy, managerial support, and developmental opportunities, which have been shown to lower burnout (Bakker et al., 2003) and enhance employee engagement (Bakker & Demerouti, 2007; Bakker & Bal, 2010). Employees with more job resources tend to increase their work effort and strive to exceed their tasks, while those who lack job resources often experience reduced motivation and commitment, leading to withdrawal from work (Bakker et al., 2004). Thus, employees’ resource status plays a crucial role in explaining their work behavior. In the context of overqualification, opportunities for development are particularly essential compared to other job resources. Opportunities for development are defined as organizationally designed job tasks that offer employees growth and development (Bakker & Bal, 2010). Overqualified employees can utilize these opportunities to fulfill their needs to apply expertise and realize personal value (Ma et al., 2021). When employees perceive strong developmental support from their organizations, they tend to report reduced emotional fatigue (Jawahar, 2012) and show greater willingness to exceed formal job requirements (Ma et al., 2021). Such developmental experiences can function as psychological buffers, lessening the negative emotional impact of perceived overqualification and thereby lowering frustration levels.

Based on COR theory, the loss of one type of resource can be compensated for by another resource of equal value (Hobfoll, 2001). For example, work-related resources can offset resource depletion resulting from family conflicts (Hirsch & Rapkin, 1986). Similarly, the perceived threat of resource depletion caused by perceived overqualification can be alleviated by job resources such as opportunities for development. In such contexts, opportunities for development provide overqualified employees with new avenues to utilize their surplus resources, thereby breaking the constraints on resource utilization and effectively mitigating the perceived threat of resource depletion. Specifically, in work environments with ample opportunities for development, overqualified employees tend to realize their underutilized potential (Luksyte & Spitzmueller, 2016) and undertake tasks that match their qualifications (e.g., engaging in innovative projects or cross-departmental collaboration) (Zacher et al., 2019), enabling idle resources to be reinvested into value creation. This positive experience helps compensate for the loss caused by resource underestimation and waste. It also enhances overqualified employees’ positive evaluations of their internal resources and reduces the stress associated with potential resource loss, which together may lower their sense of frustration. Conversely, a lack of opportunities for development restricts the ways in which overqualified employees can showcase their talents and strengths. This situation is associated with diminished work determination, confidence, and sense of achievement (Rego & Cunha, 2009), and may, in turn, relate to further resource waste and loss threats. In this context, overqualified employees may feel increasingly undervalued, and such feelings are likely to be linked to a greater sense of frustration. Hence, the following hypothesis is proposed:

**H3.**  *Opportunities for development moderate the relationship between perceived overqualification and frustration, such that this relationship is weaker when there are more opportunities for development compared to when there are fewer.*

Building on the above reasoning, we argue that the frustration-based pathway linking perceived overqualification to cyberloafing may appear less salient in contexts where opportunities for development are readily available. As posited by COR theory, access to a broader set of psychological or professional resources relate to more adaptive emotional responses and promotes constructive behavioral patterns at work (Hobfoll, 2001). When employees perceive sufficient opportunities for growth, they are more likely to remain engaged and resilient in the face of workplace dissatisfaction (Bakker et al., 2003; Bakker & Bal, 2010; Bakker & Demerouti, 2007). For overqualified individuals in particular, developmental opportunities function as compensatory resources that help offset emotional and cognitive strain, thus reducing the likelihood of engaging in cyberloafing. Conversely, in environments lacking such support, the persistent sense of resource underutilization may increase frustration. In this situation, overqualified employees may resort to cyberloafing to protect resource. Therefore, the following hypothesis is proposed:

**H4.**  *Opportunities for development moderate the indirect effect of perceived overqualification and cyberloafing through frustration, such that this indirect effect is weaker when there are more opportunities for development compared to when there are fewer.*

**Figure 1:**
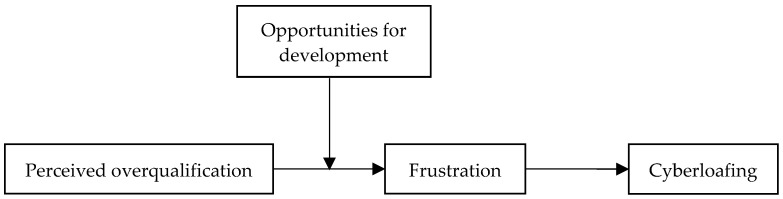
Conceptual model.

Based on the theoretical propositions outlined above, the conceptual model guiding this study is presented in Figure 1.

## 3. Methods

### 3.1. Sample and Procedures

To examine the proposed hypotheses, we employed a structured questionnaire approach to gather data from full-time employees across various industries, which includes manufacturing, finance, and real estate, with a geographical focus on Guangdong and Guangxi Provinces in China. Participants were selected based on their regular access to workplace digital devices (e.g., smartphones and computers) during working hours, ensuring that the conditions for cyberloafing were present.

Prior to data collection, human resource managers from ten companies were contacted via telephone to clarify the research objectives. Upon securing their approval, employees from different departments were invited to voluntarily participate. A total of 500 paper-based surveys were distributed and returned on-site. To ensure anonymity, no identifying personal information was recorded. A two-wave data collection strategy was designed to address the issue of common method variance. To enable pairing, respondents entered the first letter of their surname in Chinese and the last four digits of their phone number on each questionnaire.

The first wave collected data on perceived overqualification, developmental opportunities, and demographic characteristics, yielding 392 completed responses (response rate: 78.4%). Two weeks later, the second wave focused on frustration and cyberloafing, generating 389 responses (response rate: 77.8%). We excluded cases in which respondents voluntarily withdrew, exhibited random or inappropriate responses, were unable to recognize reversed items, provided missing values, or could not be matched across the two-stage questionnaires. Following this exclusion procedure, 301 valid paired responses were retained, corresponding to a usable response rate of 60.2%.

Among the final sample, 53.2% were male and 46.8% female, the average age was 36.22 years (SD = 9.90), most of the participants held a bachelor’s degree (53.8.2%), and approximately 50.8% were married (see Table 1 for detailed demographics).

### 3.2. Measures

Before we distributed the questionnaire, we translated the mature English questionnaire into Chinese following the back-translation suggested by Brislin (1980). All measurement instruments were measured using a 5-point Likert scale (from 1 = “strongly disagree” to 5 = “strongly agree”).

#### 3.2.1. Perceived Overqualification (Time 1)

Perceived overqualification was measured using the nine-item Scale of Perceived Overqualification (SPOQ) developed by Maynard et al. (2006). A sample item is “My job requires less education than I have”. Cronbach’s alpha for this scale was 0.90.

#### 3.2.2. Frustration (Time 2)

Frustration was measured using the three-item scale developed by Peters et al. (1980). A sample item is “Trying to get this job done was a very frustrating experience”. Cronbach’s alpha for this scale was 0.81.

#### 3.2.3. Opportunities for Development (Time 1)

Opportunities for development was measured using the three-item scale developed by Bakker and Bal (2010). A sample item is “My work offered me the opportunity to learn new things”. Cronbach’s alpha for this scale was 0.82.

#### 3.2.4. Cyberloafing (Time 2)

Cyberloafing was measured using the nine-item scale developed by S. Andel et al. (2022). A sample item is “Spend time sending/looking at non-work-related messages, photos, and videos”. Cronbach’s alpha for this scale was 0.91.

#### 3.2.5. Control Variables (Time 1)

This study incorporates gender, age, education level, and marital status as control variables, given that previous research has demonstrated that these variables are significantly associated with the occurrence of cyberloafing (Andreassen et al., 2014; Lim & Chen, 2012; Sarıoğlu Kemer & Dedeşin Özcan, 2021; J. Wu et al., 2020). In this study, gender (1 = male, 0 = female) and marital status (1 = Married, 0 = Single) were dummy coded. Education level was categorized into three groups, with dummy variables created for bachelor’s degree and master’s degree or above, using college degree or below as the reference category.

#### 3.2.6. Data Analysis

We utilized Mplus 8.8 software to analyze discriminant validity and convergent validity. Furthermore, we employed SPSS 27.0.1 software to conduct descriptive statistical analyses, correlation analyses, and reliability assessments of the scales and used the PROCESS macro to test the hypothesized models, including moderation and mediated moderation models. To estimate the respective effects, we applied bootstrapping with 5000 resamples and a 95% confidence interval.

## 4. Results

### 4.1. Common Method Bias

In line with best practices for minimizing common method variance (Podsakoff et al., 2012), a two-wave data collection strategy was adopted over the course of one month, with a two-week interval between stages. Previous studies have validated the use of such a temporal gap to address issues of bias and establish temporal precedence (M. J. Zhang et al., 2016; J. Khan et al., 2022b; Zhu et al., 2024). Furthermore, reversed items were designed in distributed questionnaires, which have been found effective in excluding careless respondents and mitigating potential common method bias (Podsakoff et al., 2012). Finally, the four measured variables were combined into a single factor, but the one-factor fitting index (χ^2^ = 1985.28, df = 252, NFI = 0.45, IFI = 0.48, TLI = 0.43, CFI = 0.48, SRMR = 0.16, RMSEA = 0.15) was suboptimal (see Table 2), indicating that there is no significant impact of common method bias on the research outcome.

### 4.2. Confirmatory Factor Analysis

To assess the distinctiveness of the latent constructs, we performed a confirmatory factor analysis (CFA) in Mplus. Model fit indices are summarized in Table 2. Among all tested configurations, the hypothesized four-factor model demonstrated the most satisfactory fit, with fit statistics indicating strong model adequacy (χ^2^ = 350.25, df = 246, NFI = 0.90, IFI = 0.97, TLI = 0.97, CFI = 0.97, SRMR = 0.04, RMSEA = 0.04). Nested comparisons revealed that this four-factor solution significantly outperformed all alternative specifications (see Table 2 for full comparisons). These findings support the discriminant validity of the constructs, allowing them to be treated as conceptually independent variables in subsequent analyses.

Next, to estimate the consistency and convergence of the latent constructs, Mplus was adopted to examine convergent validity. Based on prior research, the convergent validity requires loadings above 0.5 for each item, a composite reliability (CR) exceeding 0.7, and an average variance extracted (AVE) greater than 0.5 (Hair et al., 2014). In Table 3, all item loadings exceeded 0.5. Perceived overqualification (AVE = 0.50, CR = 0.90), frustration (AVE = 0.59, CR = 0.81), opportunities for development (AVE = 0.60, CR = 0.82), and cyberloafing (AVE = 0.54, CR = 0.91) all demonstrated good convergent validity, indicating that the questionnaire items effectively captured the measured variables.

### 4.3. Descriptive Statistics and Correlations

Table 4 displays the means, standard deviations, and correlation coefficients for each variable. It can be observed that perceived overqualification was significantly positively related to cyberloafing (r = 0.27, *p* < 0.01) and frustration (r = 0.17, *p* < 0.01). Additionally, frustration was significantly positively related to cyberloafing (r = 0.29, *p* < 0.01). These results provide initial support for our Hypotheses 1, 2, and 3.

### 4.4. Hypothesis Testing

Table 5 presents the results of the linear regression analysis. Hypothesis 1 predicts that perceived overqualification is positively related to cyberloafing. As shown in Model 3, perceived overqualification has a significant positive effect on cyberloafing (B = 0.26, *p* < 0.001), thus supporting Hypothesis 1.

Hypothesis 2 asserts that perceived overqualification has an indirect effect on cyberloafing via frustration. The results shows that perceived overqualification is positively related to cyberloafing (B = 0.22, *p* < 0.01; Model 1), and frustration is positively related to cyberloafing. The results, as shown in (B = 0.22, *p* < 0.001; Model 4). In Model 5, the results indicate that after accounting for frustration, the effect of perceived overqualification on cyberloafing (B = 0.22, *p* < 0.001) became weaker, while the effect of frustration on cyberloafing (B = 0.19, *p* < 0.001) remained significant, suggesting partial mediation. The bootstrapping results presented in Table 6 reveal that the indirect effect of perceived overqualification on cyberloafing through frustration is significant (B = 0.04, SE = 0.02), with a 95% confidence interval [0.01, 0.09] that does not include zero, indicating statistical significance. Therefore, Hypothesis 2 is supported.

**Figure 2:**
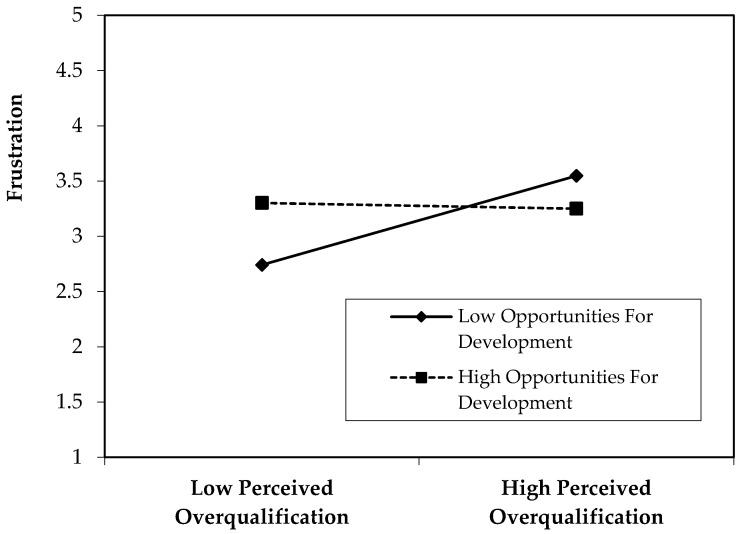
Moderating effect of opportunities for development on the relationship between perceived overqualification and frustration.

Hypothesis 3 predicts that opportunities for development moderates the relationship between perceived overqualification and frustration, such that this relationship is weaker when there are more opportunities for development compared to when there are fewer. As shown in Model 2 of Table 5, the regression coefficient for the interaction term is negative and significant (B = −0.24, *p* < 0.001), supporting Hypothesis 3. Figure 2 displays the interaction plot based on values plus or minus one standard deviation from the mean of opportunities for development. Specifically, Table 7 demonstrates that when opportunities for development is low, the effect of perceived overqualification on frustration is strong and significant (B = 0.64, *p* < 0.001, 95% CI [0.36, 0.92]), whereas when opportunities for development is high, the effect is not significant (B = −0.02, *p* > 0.05, 95% CI [−0.21, 0.18]). These results demonstrate that opportunities for development buffer the positive relationship between perceived overqualification and frustration, thus providing support for Hypothesis 3.

Finally, to test the moderated mediation, this study conducted a conditional indirect effect bootstrapping analysis (see Table 8). The statistical results suggest that the indirect effect of perceived overqualification on cyberloafing via frustration was stronger and significant when opportunities for development was low (B = 0.12, SE = 0.04, 95% CI [0.06, 0.20]) and weaker and not significant when opportunities for development was high (B = −0.00, SE = 0.02, 95% CI [−0.04, 0.04]). Moreover, the moderated mediation index was significantly negative (B = −0.05, SE = 0.02, 95% CI [−0.08, −0.02]). Therefore, Hypothesis 4 is supported.

## 5. Discussion

### 5.1. Theoretical Implications

This study makes three theoretical contributions. First, this study presents a novel theoretical framework for understanding why overqualified employees engage in cyberloafing. The majority of previous investigations are based on equity theory and person-job fit theory and presume that cyberloafing is a reflection of resistance to organizational injustice (Cheng et al., 2020; J. Khan et al., 2022a) or disengagement from an inappropriate assignment (S. Andel et al., 2022). This study takes the proactive angle of resource conservation as highlighted by COR theory (Hobfoll, 1989; Hobfoll et al., 2018). To be precise, ignorance and underutilization of valuable knowledge, skills, and credentials evoke a sense of threat of depreciation among overqualified employees when such resources have been self-earned but are considered useless by the employer (Jiang et al., 2024; Shang et al., 2024; X. Wu & Ma, 2023). Consequently, cyberloafing becomes a proactive resource recovery behavior for them to realize self-resource mobilization, which preserves existing resources and halts any further dissipation (Peng et al., 2023; X. Zhang et al., 2024). Therefore, this research offers a refined view on the internal resource evaluation that leads overqualified employees to the internet for non-work use, extends COR theory to show how cyberloafing may be an adaptive response adopted to deal with current or prospective resource drain, and improves understanding of the anticipatory worries that employees have about resource loss and their corresponding strategies.

Secondly, this study delves into the mechanisms that connect the phenomenon of overqualification with cyberloafing from the standpoint of resource conservation. While cyberloafing has been considered a sort of work deviance, it is different from other deviant behaviors since it provides employees with a chance to escape from their tasks and restore resources (S. A. Andel et al., 2019; Tsai, 2023). Previous research that informs the link between overqualification perception and cyberloafing focused on negative work experiences, which are associated with emotional responses of anger (J. Khan et al., 2022a) and boredom (S. Andel et al., 2022), and disregarded cyberloafing as a resource-renewing behavior. COR theory assumes that overqualified employees have sufficient resources, according to Guo et al. (2024), but job restrictions limit the use of their abilities, and such preventable constraints lead to frustration. This negative emotional state uniquely conveys resource underutilization and dramatically depletes cognitive and emotional resources, which turns the assumption about resource loss resulting from overqualification into actual depletion (González-Gómez & Hudson, 2024). In this situation, cyberloafing serves as an adaptive strategy for overqualified employees to restore resources. Thus, more broadly, frustration not only causes cyberloafing among overqualified employees but also explains why such behavior functions as a restorative mechanism for them.

Thirdly, this research also examines the situational variables of job resources and how they contribute to reducing the adverse impact of perceived overqualification on frustration and its mediation effect on cyberloafing. This, therefore, enhances the theoretical base on job resources and perceived overqualification. Answering the call to integrate organizational context variables as boundary conditions (Shang et al., 2024; X. Wu & Ma, 2023), this study asserts that opportunities to grow lessen overqualified workers’ fear of near-job resource loss caused by their underutilization of resources. In an environment with high opportunities for development, overqualified employees can reach training and career advancement targets and eventually break the barrier that overqualification imposes on resource utilization. In COR theory, workers’ opportunities to grow act as fuel for resource recovery and help decrease frustration caused by their utilization of resources. This prevents employees from entering a cycle of resource depletion. Thus, overqualified employees have a low probability of cyberloafing for compensatory recovery. Thus, opportunities for development offer a novel pathway for mitigating the negative response of perceived overqualification and provide fresh insights into how organizations can unlock the potential of their overqualified workforce.

### 5.2. Practical Implications

Drawing from the findings, this study outlines three actionable recommendations to help organizations mitigate the adverse effects associated with perceived overqualification. First, as overqualification may prompt behaviors such as cyberloafing, firms should consider refining both recruitment and talent management practices to proactively address potential mismatches. During hiring, HR professionals could offer realistic job previews that convey the scope, responsibilities, and developmental trajectory of the role to set accurate expectations. Moreover, a holistic selection framework should be adopted to assess the compatibility between applicants’ qualifications and the actual demands of the position, thereby reducing the likelihood of employees perceiving themselves as overqualified.

Second, this study reveals that frustration mediates the relationship between perceived overqualification and cyberloafing. Frustration in real-life situations mainly arises from obstacles in achieving goals or insufficient material rewards. Organizations should actively communicate with overqualified employees to understand their thoughts and needs, provide material or non-material incentives that align with their employees’ contributions, and help reduce their employees’ sense of frustration and other negative feelings.

Third, this study’s findings indicate that opportunities for development can mitigate negative emotions (i.e., frustration) caused by perceived overqualification and reduce employees’ engagement in deviant behaviors (i.e., cyberloafing). In this regard, organizations can expand the application scenarios of overqualified employees’ knowledge, skills, and experience by assigning them challenging tasks, involving them in cross-departmental projects, and providing personalized career development paths. Furthermore, organizations should grant overqualified employees greater decision-making authority and expanded job responsibilities, enabling their qualifications to be leveraged at a higher level. Concurrently, by offering promotion opportunities or lateral development paths, organizations can help these employees discover new avenues for professional growth.

### 5.3. Limitations and Directions for Future Research

This study has several important limitations. First, data were obtained through self-report measures, which may introduce common method bias. For instance, research data on cyberloafing might have been underreported due to participants’ concerns about social desirability. Although the use of a two-wave data collection design helped reduce this issue to some extent, future research could address it more rigorously by adopting multi-source data. For example, instead of relying on self-reported surveys, researchers might employ digital monitoring tools to objectively track non-work-related internet usage during work hours, thereby reducing bias related to shared measurement methods.

Second, this study was conducted in Guangdong and Guangxi Provinces due to the research team’s existing collaborations with local companies, which facilitated data access. These provinces represent a variety of industries and organizational forms, making them suitable for examining the impact of perceived overqualification on cyberloafing. However, we acknowledge that the regional focus may limit the generalizability of our findings, as the economic, cultural, and organizational characteristics of these provinces may differ from those of other regions. Future research in other regions could help broaden the applicability of the results.

Third, this study examined frustration, which is an emotional variable, as a mediator. However, frustration partially mediates the relationship between perceived overqualification and cyberloafing, and its mediating effect was determined to be statistically significant but modest in hypothesis testing. These results hint that there might be a more plausible underlying mechanism to explain the impact of perceived overqualification on cyberloafing. Thus, future research could incorporate cognitive variables (e.g., self-determination, self-concept, role identity) to further explain why perceived overqualification leads to cyberloafing. Additionally, future research examining the comprehensive dual-path theoretical framework could enhance our understanding of the psychological process linking perceived overqualification and cyberloafing.

Lastly, this study only considered opportunities for development as a job resource when examining moderating effects. Future research could focus on the effects of other job resources (e.g., autonomy, supervisory coaching) on perceived overqualification and subsequent work behaviors, thereby expanding the application scenarios of job resources in management research. In particular, Hobfoll (2001) identified 74 key resources that organizations can provide to employees, including essential work tools and positive challenging daily tasks. Future studies could investigate how different types of organizational resources affect the work behaviors and attitudes of overqualified employees, exploring additional boundary conditions.

## 6. Conclusions

Drawing on COR theory, this study constructed a moderated mediation model to empirically examine how and when perceived overqualification influences employee cyberloafing. This study yields two major research findings. First, perceived overqualification increases employees’ cyberloafing by triggering frustration. Overqualification represents a waste of resources, creating restrictive conditions for resource utilization at work, which leads to frustration; employees experiencing frustration may engage in cyberloafing to relieve the replenish resources depleted by this negative emotion. Second, the buffering role of developmental opportunities was confirmed, as their presence significantly weakened both the emotional strain associated with perceived overqualification and its downstream behavioral expression in the form of cyberloafing. These results imply that, under conditions where developmental support is limited, employees are more likely to experience frustration stemming from perceived overqualification, which in turn increases the likelihood of cyberloafing. By uncovering this dual-stage moderating effect, this study adds nuance to our understanding of the mechanisms and contextual boundaries that shape how overqualification influences deviant workplace behavior.

## Figures and Tables

**Table 1:** Descriptive statistics of sample characteristics.

Characteristics	Frequency	Percentage (%)
Gender		
Male	141	53.2
Female	160	46.8
Age		
Below the age of 26	54	17.9
26–30	56	18.6
31–35	43	14.3
36–40	52	17.3
Above the age of 40	96	31.9
Education level		
College degrees or lower	56	18.6
Bachelor’s degrees	162	53.8
Master’s degrees or beyond	83	27.6
Marital status		
Single	148	49.2
Married	153	50.8

Note: N = 301.

**Table 2:** The results of confirmatory factor analyses.

Measurement Model	χ^2^	df	NFI	IFI	TLI	CFI	SRMR	RMSEA	Δχ^2^/(Δdf)
Four-factor model	350.25	246	0.90	0.97	0.97	0.97	0.04	0.04	
Three-factor model	665.75	249	0.82	0.88	0.86	0.88	0.08	0.08	105.17 ***
Two-factor model	1670.35	251	0.54	0.58	0.53	0.58	0.15	0.14	264.02 ***
One-factor model	1985.28	252	0.45	0.48	0.43	0.48	0.16	0.15	272.51 ***

Note: Four-factor model (perceived overqualification, frustration, opportunities for development, and cyberloafing); three-factor model (combined perceived overqualification with frustration); two-factor model (combined perceived overqualification, frustration, and cyberloafing); one-factor model (combines all research variables together); N = 301, *** *p* < 0.001.

**Table 3:** Item loadings.

Construct	Estimate	SE	AVE	CR
Perceived overqualification				
1	0.70	0.03	0.50	0.90
2	0.75	0.03		
3	0.69	0.03		
4	0.71	0.03		
5	0.66	0.04		
6	0.73	0.03		
7	0.76	0.03		
8	0.67	0.04		
9	0.73	0.03		
Frustration				
1	0.80	0.03	0.59	0.81
2	0.71	0.04		
3	0.81	0.03		
Opportunities for development				
1	0.75	0.04	0.60	0.82
2	0.80	0.03		
3	0.78	0.03		
Cyberloafing				
1	0.72	0.03	0.54	0.91
2	0.74	0.03		
3	0.71	0.03		
4	0.71	0.03		
5	0.75	0.03		
6	0.78	0.03		
7	0.75	0.03		
8	0.73	0.03		
9	0.72	0.03		

Note: N = 301.

**Table 4:** Descriptive statistics and correlations.

Variables	Mean	SD	1	2	3	4	5	6	7	8	9	10
1. Gender	0.47	0.50										
2. Age	36.22	9.90	−0.00									
3. College degrees or lower	0.19	0.39	−0.02	0.03								
4. Bachelor’s degrees	0.54	0.50	0.04	−0.03	−0.52 **							
5. Master’s degrees or beyond	0.28	0.45	−0.03	0.01	−0.30 **	−0.67 **						
6. Marital status	0.51	0.50	−0.06	0.58 **	−0.01	−0.02	0.03					
7. Perceived overqualification	3.90	0.82	0.00	0.06	−0.03	−0.13 *	0.16 **	0.02	(0.90)			
8. Cyberloafing	3.90	0.87	0.11	0.18 **	0.01	−0.12 *	0.12 *	0.08	0.27 **	(0.91)		
9. Frustration	3.45	1.09	0.02	0.11	0.03	−0.04	0.02	0.13 *	0.17 **	0.29 **	(0.81)	
10. Opportunities for development	3.66	1.09	0.01	−0.09	0.00	−0.14 *	0.15 **	−0.07	0.10	0.06	0.05	(0.82)

Note: N = 301; Cronbach’s alphas are displayed in parentheses; * *p* < 0.05, ** *p* < 0.01.

**Table 5:** Results of hypothesis testing.

Variable	Frustration				Cyberloafing					
	M1		M2		M3		M4		M5	
Control Variable	B	SE	B	SE	B	SE	B	SE	B	SE
Gender	0.06	0.12	0.04	0.12	0.19 *	0.10	0.18	0.10	0.18	0.09
Age	0.00	0.01	0.01	0.01	0.02 *	0.01	0.02 *	0.01	0.01 *	0.01
Bachelor’s degrees	−0.09	0.17	−0.08	0.16	−0.1	0.13	−0.09	0.13	−0.85	0.13
Master’s degrees or beyond	−0.09	0.19	−0.12	0.18	0.09	0.14	0.17	0.14	0.11	0.14
Marital status	0.22	0.15	0.20	0.15	−0.03	0.12	−0.09	0.12	−0.07	0.11
Independent Variable										
Perceived overqualification	0.22 **	0.08	0.23 **	0.08	0.26 ***	0.06			0.22 ***	0.06
Mediator										
Frustration							0.22 ***	0.04	0.19 ***	0.04
Moderator										
Opportunities for development			0.06	0.06						
Interaction										
Perceived overqualification × Opportunities for development			−0.24 ***	0.07						
F	2.37 *		3.42 ***		6.54 ***		7.53 ***		8.68 ***	
R^2^	0.05		0.09		0.12		0.13		0.17	
ΔR^2^			0.04		0.03		0.01		0.04	

Note: N = 301; * *p* < 0.05; ** *p* < 0.01; *** *p* < 0.001. The regression coefficients (B) are unstandardized coefficients, and SE represents the standard errors.

**Table 6:** Results of total, direct, and indirect effects of perceived overqualification and cyberloafing via frustration.

	Coefficient	SE	Bootstrapped Confidence Interval (95%)	
			LLCI	ULCI
Total effect	0.26	0.06	0.14	0.37
Direct effect	0.22	0.06	0.10	0.33
Indirect effect	0.04	0.02	0.01	0.09

Note: N = 301.

**Table 7:** Conditional effects of perceived overqualification and frustration at different levels of opportunities for development.

	Coefficient	SE	LLCI (95%)	ULCI (95%)
Low opportunities for development (M − 1SD)	0.64	0.14	0.36	0.92
High opportunities for development (M + 1SD)	−0.02	0.10	−0.21	0.18

Note: N = 301.

**Table 8:** Conditional indirect effects of perceived overqualification and cyberloafing via frustration at different levels of opportunities for development.

	Coefficient	SE	LLCI (95%)	ULCI (95%)
Low opportunities for development (M − 1SD)	0.12	0.04	0.06	0.20
High opportunities for development (M + 1SD)	−0.00	0.02	−0.04	0.04
Moderated mediation index	−0.05	0.02	−0.08	−0.02

Note: N = 301.

## Data Availability

The datasets analyzed in the current study are available from the corresponding author upon reasonable request.
